# Diet, β-glucocerebrosidase deficiency, and Parkinson’s disease

**DOI:** 10.1016/j.jlr.2024.100689

**Published:** 2024-10-28

**Authors:** James A. Shayman

**Affiliations:** Department of Internal Medicine, University of Michigan, Ann Arbor, MI, USA

Gaucher disease (GD) is a lysosomal storage disorder arising from an inherited loss in activity of the lysosomal hydrolase glucosylceramidase beta 1 GBA1. The clinical manifestations of non-neuronopathic GD type 1 include hepatosplenomegaly, anemia, thrombocytopenia, and bone disease. These complications are clinically managed by therapeutic options such as enzyme replacement and oral substrate reduction therapy. The treatment of long-term complications of GD, including pulmonary fibrosis, multiple myeloma, and notably Parkinson’s disease (PD), has been more difficult. The association between GD and PD has received considerable attention over the last fifteen years based on the observation that variants in *GBA1* are the most common genetic risk factor for PD and Lewy body dementia (1). Establishing the pathogenic mechanism that links variants in GBA1 to PD has been challenging and unsuccessful in part due to the low penetrance of PD among all patients with GD type 1 and the long time to outcome from diagnosis of GD to clinical expression of PD.

Some of the proposed mechanisms linking the development of PD in association with GBA1 variants and loss of glucocerebrosidase activity include proteostatic stress secondary to misfolded GBA resulting in decreased α-synuclein degradation (2), the accumulation of lipid substrates including glucosylceramide and glucosylsphingosine which physically interact with α-synuclein (3), alterations in lysosome-mitochondrial contacts secondary to defective modulation of TCB1D15 (4), a tethering protein, and competition between misfolded GBA and other substrates for chaperone-mediated autophagy (5). While there is experimental support for each of these mechanisms, definitive experimental and clinical proof in support of any individual theory has remained elusive.

The diagnosis of GD is primarily established with enzymatic assays demonstrating a reduced activity of GBA1. the glucocerebrosidase. In the current issue of the Journal of Lipid Research, Bannink and coworkers report the identification of more specific fluorogenic substrates for the measurement of GBA1 activity for the diagnosis of GD (6). This endeavor was motivated by recognition that two non-lysosomal β-glucocerobrosidases, GBA2 and GBA3, are present in cells which also recognize the substrate 4-methylumbelliferyl-β-D-glucopyranoside. They elegantly identified the fluorogenic compound 6-O-alkyl-4-methylumbelliferyl-β-D-glucopyranoside as a substrate with high specificity toward GBA1 when compared to GBA2 and GBA3.

In pursuing this work these investigators noted that a subclass of phytosterols, notably campesterol, β-sitoserol, and sigmasterol, structurally resemble 6-O-alkyl-4-methylumbelliferyl-β-D-glucopyranoside with acyl groups at C-6 raising the question as to whether 6-O-acyl-glycosyl-phytosterols might accumulate in tissues of GD patients, including brain (Fig. 1). In preliminary data they report increases in 6-O-acyl-glycosylphytosterols in GD spleens. Because these tissues were obtained from an older collection of resected spleens (a procedural no longer recommended for the management of GD), the clinical data including presence of PD, genotyping, dietary and drug history, and analyses of other tissues was limited. Thus, these preliminary findings will require confirmation in a better controlled study. The removal of 6-O-acyl-glucose from 4-methylumbelliferone and sterols including cholesterol, campesterol and sitosterol by GBA raises the hypothesis that ingested phytosterols, including sitosterol-β-D-glycoside and β-sitosterol are causally connected to the development of PD in the setting of GBA deficiency.

**Fig. 1:**
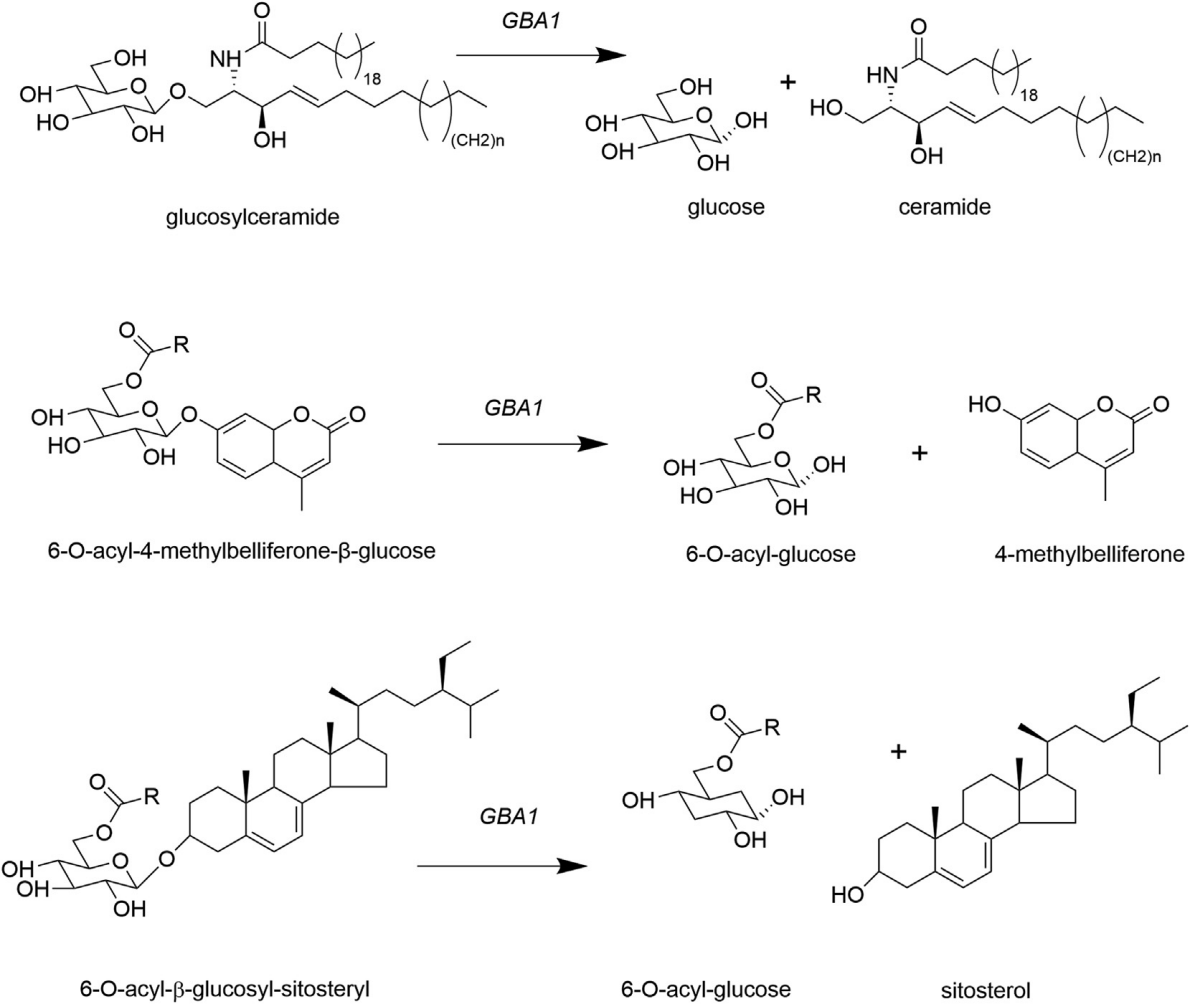
Substrates for lysosomal β-glucocerebrosidase (GBA1). Glucosylceramide, the product of UDP-glucose and ceramide, is the endogenous substrate for GBA1. 6-O-Acyl-4-methylbelliferone-β-glucose is a GBA1-specific substrate for the measurement of lysosomal glucocerebrosidase activity. 6-O-Acyl-β-glucosyl-sitosteryl is an example of a dietary ergosterol-based acyl-glucoside proposed to be a substrate for GBA1.

There is biological plausibility to this hypothesis. The low penetrance of PD in classic GD and occurrence in carriers of GBA variants is consistent with the contribution of non-genetic modifiers such as diet that would confer risk for the development of PD. The dietary consumption of ergosterols is common and ubiquitous. Plant sterols are popular dietary supplements and have been promoted as a means for lowering cholesterol levels. As pointed out by the authors, feeding sitosterol-β-D-glycoside to rats results in a PD-like phenotype (7). Sitosterol-β-D-glycoside consumption has been implicated in the high rate of PD in Guam (8). Acyl-phytosterol glycosides are present in the lipidome of spelt, wheat, coffee, and legumes but the characterization of these compounds has been limited to date (9).

Historically, the pursuit of pathogenic mechanisms of LSDs has been directed to either the sphingolipid substrates that accumulate in lysosomes or to the properties of the defective enzyme or transporter. It is not surprising then that much of the work on the PD/GD connection has focused on the potential roles of glucosylceramide, glucosylsphingosine, or misfolded GBA. Lysosomal hydrolases, however, tend to have substrate recognition that extends well beyond the endogenous proteins, lipids, DNAs, or RNAs This recognition includes endogenous substrates that are post-translationally modified or of translated proteins from variants that affect folding or solubility such as with tauopathies. In addition, exogenous substrates that are trafficked to the lysosome, for example from microbial defense, are metabolized for antigen presentation. It is not unreasonable to consider that there might be pathological consequences to impaired lysosomal catabolism of these substrates. This is exemplified by lysosomal phospholipase A2 (PLA2G15), a broad specificity phospholipase that hydrolyzes fatty acyl groups from endogenous glycerophospholipids, oxidized phospholipids, and exogenously trafficked microbial lipids (10).

There would be obvious benefits that would follow from establishing a role for acyl-glycoside sterols in the development of PD in the Gaucher population. First, these sterols might serve as biomarkers for assessing the long-term risk of developing PD. Second, specific *GBA* variants may be demonstrated to be more closely correlated with greater impairment in the catabolism of the acylated sterols identifying a subgroup of GD patients at higher risk. Third, better, more rational treatment strategies could be developed for the prevention of PD in this population. Dietary restrictions that limit consumption of these sterols would be considered. In addition, pharmacological treatment strategies that favor induction of increased GBA activity (gene therapy or chaperones) would be prioritized over those that seek to prevent substrate accumulation of endogenous sphingolipids.

While demonstrating a causal link between 6-O-acyl-glycosyl-phytosterols and Gaucher-associated PD will be challenging, the insights gained and follow-on therapeutic options more than justify such an endeavor.

## Data availability

All supporting data are provided within the manuscript, supplementary data and supplementary tables.

## Conflict of interests

The authors declare that they have no conflicts of interest with the contents of this article.
